# Integrin CD11b attenuates colitis by strengthening Src-Akt pathway to polarize anti-inflammatory IL-10 expression

**DOI:** 10.1038/srep26252

**Published:** 2016-05-18

**Authors:** Xiang Hu, Chaofeng Han, Jing Jin, Kewei Qin, Hua Zhang, Tianliang Li, Nan Li, Xuetao Cao

**Affiliations:** 1 National Key Laboratory of Medical Molecular Biology & Department of Immunology, Institute of Basic Medical Sciences, Peking Union Medical College, Chinese Academy of Medical Sciences, Beijing 100730, China; 2 National Key Laboratory of Medical Immunology & Institute of Immunology, Second Military Medical University, Shanghai, China

## Abstract

Interleukin-10 (IL-10) plays a central role in regulation of intestinal mucosal homeostasis and prevention of inflammatory bowel disease (IBD). We previously reported that CD11b^hi^ regulatory dendritic cells (DCs) can produce more IL-10, and CD11b can negatively regulate Toll-like receptors (TLRs)-induced inflammatory responses in macrophages. However whether CD11b and its signaling can control autoimmunity via IL-10 production remains unclear. Here we found that CD11b deficient (*Itgam*^−/−^) mice were more susceptible to dextran sulfate sodium (DSS)-induced colitis, with more tumor necrosis factor α (TNF-α) while less IL-10 production. CD11b inhibited nuclear factor-kappa B (NF-κB) while promoted activator protein 1 (AP-1) activation through activating sarcoma oncogene (Src), leading to decreased TNF-α while increased IL-10 production. Src interacted with and promoted c-casitas B lineage lymphoma proto-oncogene (c-Cbl)-mediated degradation of the inhibitory subunit p85 of phosphatidylinositol 3-kinase (PI3K). Importantly, Src inhibitor dasatinib aggravated DSS-induced colitis by decreasing IL-10 while increasing TNF-α *in vivo*. Therefore, CD11b promotes IL-10 production by activating Src-Akt signal pathway. An axis of CD11b-Src pathway is important in balancing homeostasis of TLR-induced pro-inflammatory and anti-inflammatory responses.

Interleukin-10 (IL-10) is an anti-inflammatory cytokine with a crucial role in preventing inflammatory and autoimmune pathologies functioning at different stages of an immune response and possibly at different anatomical locations via signal transducer and activator of transcription 3 (STAT3)^1^,^2^. IL-10 is produced by both the adaptive immune system, including T_h1_, T_h2_ and T_h17_ cell subsets, T_reg_ cells, CD8^+^ T cells and B cells, and the innate immune system, including dendritic cells (DCs), macrophages, mast cells, natural killer (NK) cells, eosinophils and neutrophils. IL-10 or IL-10 receptor (IL-10R)-deficient mice develop inflammatory bowel disease^3^, which is mainly caused by microorganisms in gut^4^. Polymorphisms in the IL-10 locus confer risk of ulcerative colitis and Crohn’s disease in human^5^,^6^,^7^. Thus, pattern-recognition receptors (PRRs) activation induced IL-10 production plays a central role in regulation of intestinal mucosal homeostasis and prevention of inflammatory bowel disease (IBD). However the recombinant IL-10 supplement therapy does not benefit IBD, which indicates that single supplement of IL-10 is not sufficient to control inflammation in IBD patients without controlling the pro-inflammatory cytokines^8^,^9^. In addition, TNF-α is chronically elevated, locally and systemically, in patients with IBD^10^. It is well known that the TNF-α plays a central role in IBD pathology and anti-TNF-α therapy has been shown to be efficacious in the treatment of ulcerative colitis and Crohn’s disease^11^. Although the anti-TNF-α therapy has been widely used, adverse side effects do occur and some patients do not respond to this therapy^12^. Therefore, signal transduction axis regulating both PRRs-activation induced pro-inflammatory and anti-inflammatory cytokines should be further investigated, hopefully identifying the promising target for drug design for the treatment of IBD^8^,^9^.

PRRs have an essential role in recognizing specific components of microorganisms and triggering innate immune responses that eliminate the invading microorganisms^13^,^14^,^15^. However, inappropriate activation of PRRs can lead to prolonged inflammation and even to autoimmune and inflammatory diseases, which partially results from increased production of inflammatory and decreased anti-inflammatory cytokines^13^,^14^,^15^. Including pro-inflammatory cytokines, such as interlukin-6 (IL-6) and TNF-α, macrophages and myeloid DCs also produce large amounts of IL-10 during pathogens infection upon TLRs, nucleotide-binding oligomerization domain 2 (NOD2), C-type lectins and DC-specific ICAM3-grabbing non-integrin (DC-SIGN) ligation^16^,^17^,^18^,^19^,^20^,^21^, which is critical to prevent over-activation of PRRs induced inflammatory responses. Thus the proper IL-10 production needs the cooperation of various signal pathways^1^. The positive or negative regulation of PRR signaling has been under intensive research. However whether there is a signal pathway controls both pro-inflammatory and anti-inflammatory cytokines remains unknown^13^,^14^,^15^. Searching the balance point of PRRs signal transduction during immune homeostasis or the unbalance point during immune pathology to produce pro-inflammatory and anti-inflammatory cytokines will be the novel question for innate response, especially during the IBD pathology.

Mac-1 (CD11b/CD18), is highly expressed on monocytes/macrophages, common dendritic cells (DCs) and regulatory DCs, which is critical for cell chemotaxis and phagocytosis^22^. CD11b deficiency breaks oral tolerance companied with increased Th17 differentiation^23^. The CD11b^hi^ DCs could inhibit T cells activation^24^, indicating CD11b can facilitate tolerance maintenance. Our previous studies show that stromal microenvironment of spleen, liver, lung can drive generation of a new population of CD11b^hi^ regulatory DCs with higher IL-10 production. These regulatory DCs could down-regulate T cell response, thus maintaining tolerance and attenuating T cell-mediated airway inflammation and hepatitis^25^,^26^,^27^,^28^. These reports outline the regulation of T cell adaptive immunity and maintenance of T cell tolerance by CD11b. We also reported that CD11b negatively regulates TLR signaling-triggered innate responses in acute infection, and CD11b-deficient mice suffer from more severe colonic damage upon acute TLR-ligation-induced sepsis with a significant increase of TNF-α production^29^. However, why the CD11b-deficient mice are prone to inflammatory autoimmune diseases has not been fully understood. Why CD11b^high^ regulatory DCs produces high amounts of IL-10 and why CD11b-deficient mice suffer more colonic damage upon acute TLR-ligation induced sepsis remain to be elusive.

Following our preliminary observation that CD11b-deficient mice are more susceptible to DSS-induced colitis, in this study, we uncovered the polarizing role and the underlying mechanisms of CD11b in promoting TLR-triggered IL-10 production in macrophages and DCs. We demonstrated that CD11b increased Src activation, which resulted in decreased NF-κB and increased AP-1 activation. This signal transduction cascade decreased TNF-α, while increased IL-10 production. We further found that Src inhibitor dasatinib aggravated DSS-induced colitis with increased pro-inflammatory cytokines while decreased anti-inflammatory cytokines *in vivo*. Therefore, CD11b promotes IL-10 production and protects mice from colitis through Src-Akt signal cascade. Our results outline a new homeostatic regulation of TLR-induced pro-inflammatory and anti-inflammatory responses by integrin-activated Src-Akt signal axis.

## Results

### CD11b-deficiency aggravates DSS-induced colitis with decrease of IL-10 production

**Figure 1:**
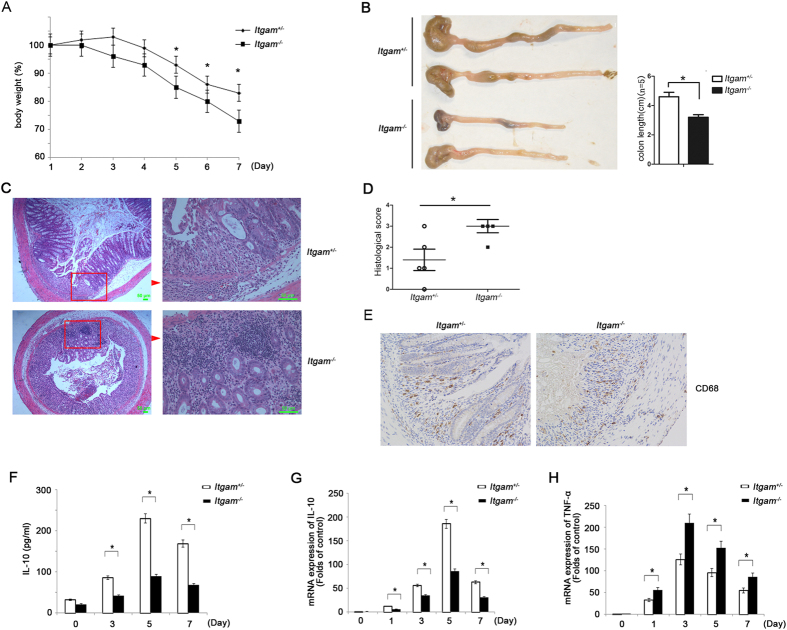
More severe DSS-induced colitis in CD11b-deficient mice. (**A**) Body weight loss (percent of initial body weight) of *Itgam*^+/−^ and *Itgam*^−/−^ mice (n = 5) after fed with 3% DSS in water. (**B,C**) Representative status and colon length data (**B**) or Hematoxylin-eosin staining (**C**) of colons from *Itgam*^+/−^ and *Itgam*^−/−^ mice (n = 5) after fed with 3% DSS in water for 7 days. Magnification: Left: ×50, Right: ×200. (**D**) The histological scores of the inflammation-associated changes in the colon from DSS-treated *Itgam*^+/−^ and *Itgam*^−/−^ mice. (**E**) Immunohistochemistry analysis of CD68 expression in the colon from DSS-treated *Itgam*^+/−^ and *Itgam*^−/−^ mice. Magnification: ×200. (**F**–**H**) ELISA of IL-10 in serum (**F**) or Q-PCR of IL-10 (**G**) and TNF-α (**H**) in the colon from *Itgam*^+/−^ and *Itgam*^−/−^ mice fed with 3% DSS in water. Data are representative of three independent experiments with similar results and presented as means ± SD. *P < 0.01.

We previously reported that regulatory dendritic cells with high expression of CD11b produced more IL-10 than common DCs, and CD11b negatively regulated TLRs-induced signals and responses in macrophages during acute inflammation^28^,^29^. However whether CD11b functions in IL-10 production remains unclear. To characterize the possible link between CD11b function and IBD, we studied CD11b-deficient mice in DSS induced colitis. A more severe colitis developed after DSS administration to CD11b-deficient (*Itgam*^−/−^) mice than their littermate heterozygous (*Itgam*^+/−^) mice with more body weight loss (Fig. 1A), shorter colon length (Fig. 1B). Furthermore, histopathology section also showed much more severe histological destruction and more inflammatory cells infiltration in the colon of *Itgam*^−/−^ mice (Fig. 1C,D). Consistent with this view, colons stained with anti-CD68 antibody showed more CD68^+^ cells infiltrating the colons of DSS-treated *Itgam*^−/−^ mice (Fig. 1E). Then we found less IL-10 in serum of *Itgam*^−/−^ mice after DSS induced colitis than their littermate control (Fig. 1F). The mRNA expression of IL-10 also decreased in the colon of *Itgam*^−/−^ mice after DSS induced colitis (Fig. 1G) compared with their littermate control. In accordance with our previous reports, the mRNA expression of TNF-α was increased in the colon of *Itgam*^−/−^ mice after DSS induced colitis (Fig. 1H). These results indicate that CD11b may prevent colitis by increasing IL-10 and decreasing TNF-α production.

### CD11b promotes TLR-triggered IL-10 production in macrophages and DCs

**Figure 2:**
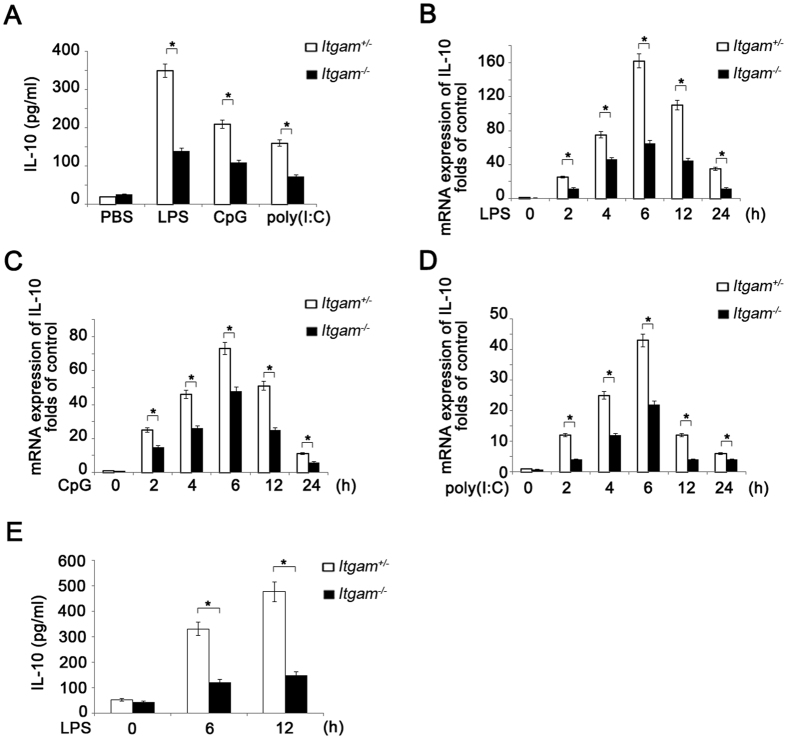
Less IL-10 production in CD11b-deficient macrophages and DCs in response to TLR ligands. (**A**) ELISA of IL-10 production in supernatant from *Itgam*^+/−^ or *Itgam*^−/−^ peritoneal macrophages (6 × 10^5^) stimulated with LPS (100 ng/ml), CpG (0.3 μM) or poly (I:C) (10 μg) for 12 h. (**B–D**) Q-PCR of IL-10 expression in *Itgam*^+/−^ or *Itgam*^−/−^ peritoneal macrophages (6 × 10^5^) stimulated with LPS (100 ng/ml), CpG (0.3 μM) or poly (I:C) (10 μg) for indicated time. (**E**) ELISA of IL-10 in supernatant from *Itgam*^+/−^ and *Itgam*^−/−^ BMDCs (8 × 10^5^) stimulated with LPS (100 ng/ml) for indicated time. Data are representative of three independent experiments with similar results and presented as means ± SD. *P < 0.01.

We previously showed that CD11b inhibited TLR-triggered inflammatory cytokines. To investigate whether CD11b can still promote the anti-inflammatory cytokine IL-10 production, TLR-ligation induced IL-10 production in the peritoneal macrophages was tested. In response to lipopolysaccharide (LPS), the synthetic RNA duplex poly (I:C) or the oligodeoxynucleotide (ODN) CpG, CD11b-knockout homogeneous (*Itgam*^−/−^) peritoneal macrophages produced less IL-10 (Fig. 2A) than CD11b-knockout heterogeneous control (*Itgam*^+/−^) macrophages. Similar results were observed between *Itgam*^+/+^ and *Itgam*^−/−^ peritoneal macrophages (supplementary Fig. 1). In order to exclude the possibility that the decrease of TLR-triggered IL-10 production may be due to the decreased survival of *Itgam*^−/−^ macrophages, we also tested the apoptosis of *Itgam*^+/−^ and *Itgam*^−/−^ peritoneal macrophage. CD11b deficiency did not affect the apoptosis of peritoneal macrophages (supplementary Fig. 2). We also detected less IL-10 mRNA in *Itgam*^−/−^ peritoneal macrophages than in *Itgam*^+/−^ upon TLR4, TLR9 and TLR3 activation (Fig. 2B–D). As mucosal DCs express markedly increased TLR2and TLR4 levels in the context of Crohn’s disease and ulcerative colitis^30^,^31^, LPS-induced IL-10 production in BMDCs was also tested. In accordance with CD11b deficiency phenotype in macrophages, *Itgam*^−/−^ BMDCs also produced less IL-10 after stimulation (Fig. 2E). And the apoptosis level of *Itgam*^−/−^ BMDCs was similar to that of *Itgam*^+/−^ BMDCs (supplementary Fig. 3). These results indicate that CD11b promotes TLR-signal induced IL-10 production.

### CD11b polarizes IL-10 production by promoting Src-Akt activation

**Figure 3:**
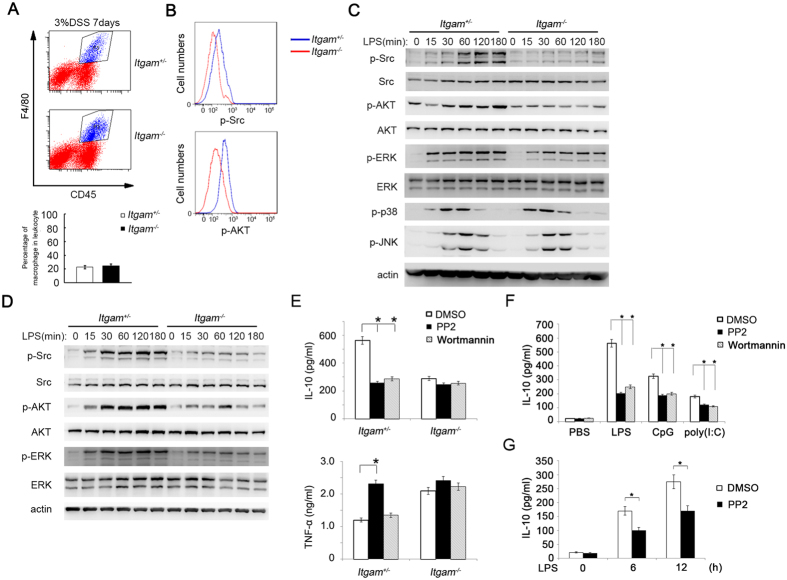
CD11b increases IL-10 production by promoting Src-Akt activation. (**A,B**) The percentage of intestinal macrophages (CD45^+^F4/80^+^) in leukocytes (CD45^+^) (**A**) and the expression level of phospho-Src and phospho-Akt of the CD45^+^F4/80^+^ cells (**B**) from the colon of the DSS-treated *Itgam*^+/−^ and *Itgam*^−/−^ mice were analyzed by FACS. A representative experiment is presented and the statistical analysis was performed by three experiments. (**C,D**) Immunoblotting of cell lysates from *Itgam*^+/−^ or *Itgam*^−/−^ peritoneal macrophages (**C**) (6 × 10^5^) and BMDCs (**D**) (8 × 10^5^) stimulated with LPS (100 ng/ml) for the indicated time with indicated antibodies. (**E**) ELISA of IL-10(up panel) and TNF-α (down panel) in the supernatant from *Itgam*^+/−^ or *Itgam*^−/−^ peritoneal macrophages (6 × 10^5^) pretreated with PP2 (10 μM) or Wortmannin (5 μM) for 30 mins and then stimulated with LPS (100 ng/ml) for 12 h. (**F**) ELISA of IL-10 in supernatant from WT peritoneal macrophages (6 × 10^5^) pretreated with PP2 (10 μM) or Wortmannin (5 μM) for 30 mins and then stimulated with LPS (100 ng/ml), poly (I:C) (10 μg/ml), or CpG-ODN (0.3 μM) for 12 h. (**G**) ELISA of IL-10 in supernatant from WT BMDCs (8 × 10^5^) pretreated with PP2 (10 μM) and the stimulated with LPS for indicated time. Data are representative of three independent experiments with similar results and presented as means ± SD. *P < 0.01.

Then to further explore how CD11b promotes TLR-induced IL-10 production, the key downstream signal pathway of CD11b was determined. In myeloid immune cells, PI3K constrains full immune cell activation by upregulating the key anti-inflammatory cytokine interleukin 10 and inhibiting of pro-inflammatory cytokines^32^,^33^. We isolated the lamina propria mononuclear cells (LPMCs), then stained with anti-CD45 and anti-F4/80 antibody, and the CD45^+^F4/80^+^ cells were gated as the intestinal macrophages (Fig. 3A up panel). The percentage of intestinal macrophages (CD45^+^F4/80^+^) in leukocytes (CD45^+^) was similar in the colon of DSS-treated *Itgam*^+/−^ and *Itgam*^−/−^ mice (Fig. 3A down panel). However, the activation of Src and Akt was impaired in *Itgam*^−/−^ intestinal macrophages, compared with that in *Itgam*^+/−^ intestinal macrophages (Fig. 3B). Accordingly, lower activation of Src and Akt was also observed in LPS-stimulated *Itgam*^−/−^ peritoneal macrophages (Fig. 3C) and BMDCs (Fig. 3D). The quantification of the phospho-Akt bands was also presented (supplementary Figs 4 and 5). The decreased activation of Src induced by LPS was in accordance with our previous reports^29^, while the activation of p38 and JNK was similar in *Itgam*^+/−^ and *Itgam*^−/−^ macrophages. Compared with *Itgam*^+/+^ control, the *Itgam*^−/−^ peritoneal macrophages also had a decreased activation of Src and Akt after LPS stimulation (supplementary Fig. 6). Similar results were concluded in *Itgam*^−/−^ peritoneal macrophages stimulated with Poly (I:C) or CpG ODN (data not shown).

To investigate whether the decreased activation of Src and Akt was involved in decreased IL-10 production in CD11b deficient mice, we used PP2 and Wortmannin to inhibit the activation of Src and Akt. Interestingly, PP2 and Wortmannin pretreatment significantly decreased LPS-induced IL-10 production in *Itgam*^+/−^ peritoneal macrophages, but did not in *Itgam*^−/−^ control (Fig. 3E up panel), which indicates that the Src and Akt are the major signal of CD11b in regulating IL-10. In addition, PP2 significantly increased TNF-α production, while Wortmannin could not in WT peritoneal macrophages, which was abolished by CD11b deficiency (Fig. 3E down panel). Above data suggest that CD11b-activated Src could both inhibit inflammatory cytokines and increase anti-inflammatory cytokine IL-10. Pretreatment of WT peritoneal macrophages with PP2 or Wortmannin decreased IL-10 protein production (Fig. 3F) and IL-10 mRNA expression level (supplementary Fig. 7) in response to LPS, Poly (I:C) and CpG ODN stimulation. PP2 pretreatment also decreased the LPS induced IL-10 production in WT BMDCs (Fig. 3G). Accordingly, other Src inhibitors (Src-I and PP1) could also decrease TLR-ligation induced IL-10 production in WT peritoneal macrophages (supplementary Fig. 8), which further confirmed the promoting function of Src on IL-10 production. These data indicate that CD11b-Src signal can both inhibit inflammatory cytokines and promote IL-10 production, leading to the polarization of anti-inflammatory cytokine production.

### Src-Akt signal cascade promotes LPS-induced IL-10 production

**Figure 4:**
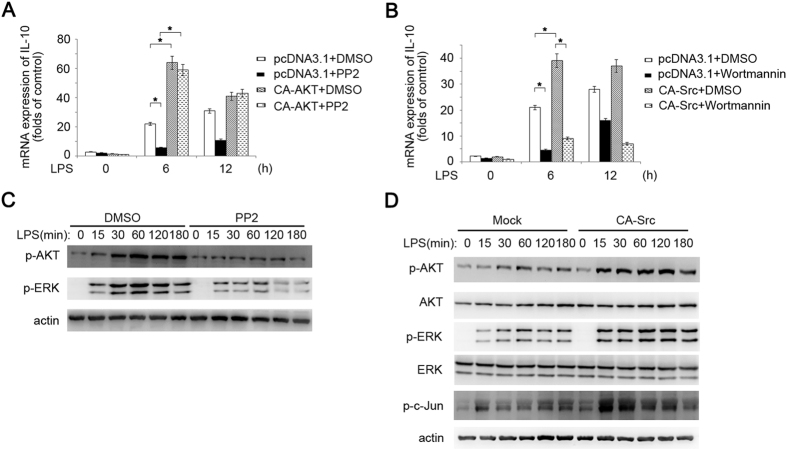
Src-Akt signal cascade promotes LPS-induced IL-10 production. (**A**) Q-PCR of IL-10 in RAW264.7 (2 × 10^5^) overexpressing constitutively active Akt (CA-Akt) or empty vector (Mock), pretreated with Src inhibitor PP2 (10 μM) for 30 mins then stimulated with LPS (100 ng/ml) for indicated time. (**B**) Q-PCR of IL-10 in RAW264.7 (2 × 10^5^) overexpressing constitutively active Src (CA-Src) or empty vector (Mock), pretreated with Wortmannin (5 μM) for 30 mins then stimulated with LPS (100 ng/ml) for indicated time. (**C**) Immunoblotting of the cell lysates from WT peritoneal macrophages pretreated with PP2 or DMSO and then stimulated with LPS (100 ng/ml) for indicated time. (**D**) Immunoblotting of the cell lysates from RAW264.7 overexpressing CA-Src or Mock and stimulated with LPS (100 ng/ml) for indicated time with indicated antibodies. Data are representative of three independent experiments with similar results and presented as means ± SD. *P < 0.01.

Then we investigate the relationship between Src-Akt signal pathway activation and IL-10 expression by the overexpression experiment in RAW264.7 cells. Overexpression of constitutively activated Akt (CA-Akt) resulted in significantly higher LPS-induced IL-10 mRNA expression than in control, and PP2 pretreatment could not reverse the Akt overexpression dependent up-regulation of IL-10 mRNA expression (Fig. 4A). Although overexpression of constitutively activated Src (CA-Src) also resulted in higher LPS-induced IL-10 mRNA expression than that in control cells, Wortmannin pretreatment could reverse the Src activation dependent up-regulation of IL-10 mRNA expression (Fig. 4B), which indicates that Src acts as the upstream of Akt.

Consistently, PP2 pretreatment also decreased LPS-triggered Akt activation (Fig. 4C, supplementary Fig. 9). However, Src activation was not affected by Wortmannin pretreatment in peritoneal macrophages (data not shown). Overexpression of CA-Src promoted Akt activation (Fig. 4D). These results suggest that Src acts as upstream of Akt in the regulation of IL-10 production by CD11b signal.

### Src-Akt signal cascade promotes AP1 through glycogen synthase kinases 3 (GSK3)

**Figure 5:**
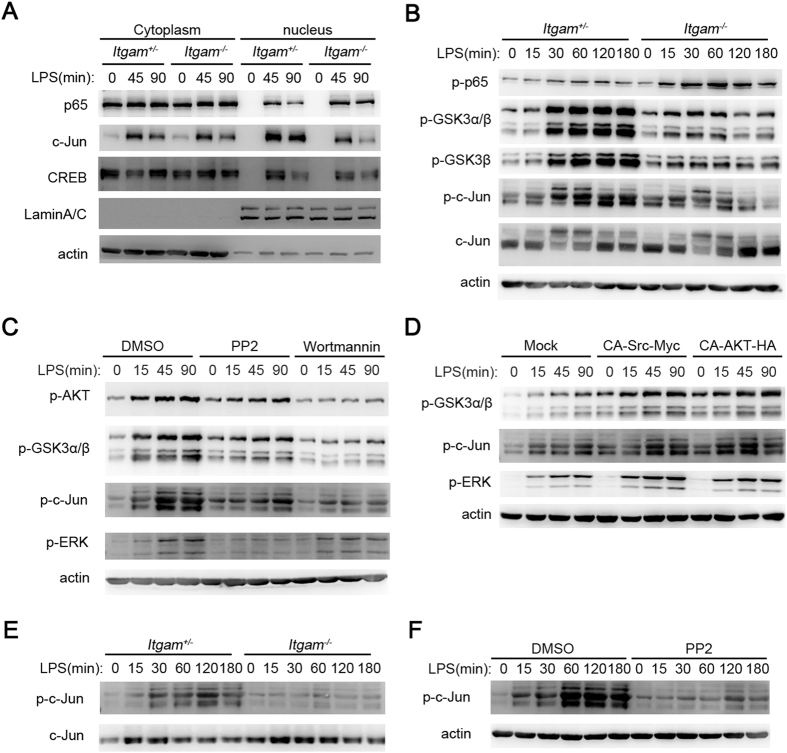
Src-Akt signal cascade promotes AP1 activation through GSK3. (**A**) Immunoblotting of cytoplasmic or nuclear extracts from *Itgam*^+/−^ or *Itgam*^−/−^ peritoneal macrophages stimulated with LPS (100 ng/ml) for indicated time with indicated antibodies. (**B**) Immunoblotting of cell lysates from *Itgam*^+/−^ or *Itgam*^−/−^ peritoneal macrophages stimulated with LPS (100 ng/ml) for indicated time with indicated antibodies. (**C**) Immunoblotting of cell lysates from WT peritoneal macrophages pretreated with PP2 (10 μM) or Wortmannin (5 μM) for 30 mins and then stimulated with LPS (100 ng/ml) for indicated time with indicated antibodies. (**D**) Immunoblotting of cell lysates from RAW264.7 cells overexpressing CA-Src or CA-Akt and then stimulated with LPS (100 ng/ml) for indicated time with indicated antibodies. (**E**) Immunoblotting of cell lysates from *Itgam*^+/−^ or *Itgam*^−/−^ BMDCs stimulated with LPS (100 ng/ml) for indicated time with indicated antibodies. (**F**) Immunoblotting of cell lysates from WT BMDCs pretreated with PP2 (10 μM) or DMSO and then stimulated with LPS (100 ng/ml) for indicated time. Data are representative of three independent experiments with similar results.

We further investigated the signal pathway in which CD11b promoted IL-10. The transciptor NF-κB, CREB and AP1 are the most important transcriptors for IL-10 in macrophages or dendritic cells upon PRRs activation^1^. More activation of p65, less activation of c-Jun and similar activation of CREB were observed in *Itgam*^−/−^ peritoneal macrophages in response to LPS stimulation, as indicated by the nuclear translocation of these transcriptors (Fig. 5A). The LPS-induced nuclear translocation of c-Fos was also decreased just as c-Jun (supplementary Fig. 10). These results indicate that the decreased IL-10 production in *Itgam*^−/−^ peritoneal macrophages involves the decreased AP-1 activation. Although the activation of NF-κB increased in *Itgam*^−/−^ peritoneal macrophages, IL-10 production decreased companied with decreased AP-1 activation as soon as upon TLR ligand stimulation (Fig. 5B), which indicates that the function of CD11b deficiency on decreasing AP-1 activation and IL-10 production is not dependent on NF-κB activation.

GSK3, the downstream kinase of Akt pathway, could inhibit AP1 activation^34^. So we next assessed the GSK3 inactivation by analyzing the phosphorylation of GSK3α/β. We found less LPS-induced phosphorylation of GSK3α/β and AP-1 in *Itgam*^−/−^ macrophages (Fig. 5B) than in *Itgam*^+/−^ control macrophages, which indicates that the inactivation of GSK3 and AP1 is involved in CD11b signaling. Consistently, less GSK3α/β and AP-1 phosphorylation were detected in WT peritoneal macrophages which were pretreated with PP2 or Wortmannin than in which were pretreated with DMSO (Fig. 5C). Higher phosphorylation of GSK3α/β and AP-1 were detected in RAW264.7 cells which overexpressed CA-Src or CA-Akt (Fig. 5D) than in control RAW264.7 cells. Similar results were also observed in BMDCs (Fig. 5E,F). These data indicate that CD11b may promote IL-10 expression via increasing AP1 activation through Src- Akt-GSK3 signal cascade.

### Src activates Akt signaling via promoting the phosphorylation and degradation of the PI3K regulatory unit p85

**Figure 6:**
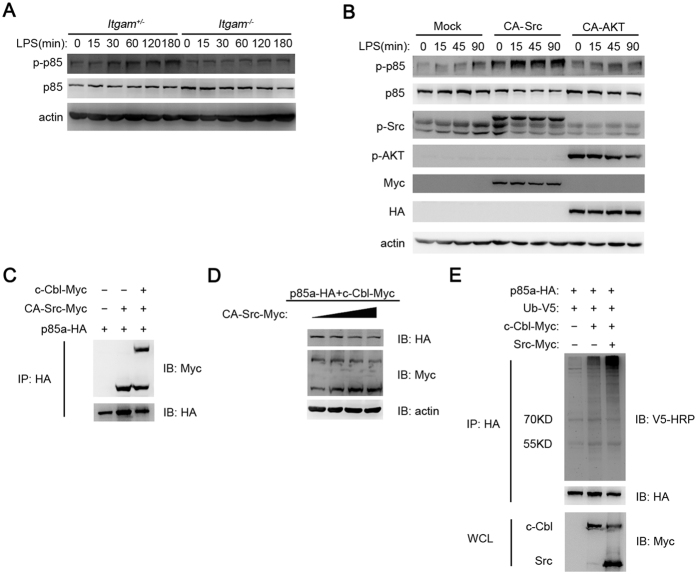
Src activates Akt signaling via promoting phosphorylation and degradation of the PI3K regulatory unit p85. (**A**) Immunoblotting of cell lysates from *Itgam*^+/−^ or *Itgam*^−/−^ peritoneal macrophages stimulated with LPS (100 ng/ml) for indicated time with indicated antibodies. (**B**) Immunoblotting of the cell lysates from RAW264.7 cells overexpressing Mock, CA-Src or CA-Akt and then stimulated with LPS (100 ng/ml) for indicated time with indicated antibodies. (**C**) Immunoblotting of immunoprecipitated production from HEK293 cells overexpressing indicated plasmids. (**D**) Immunoblotting of the cell lysates from HEK293 cells overexpressing indicated plasmids. (**E**) Immunoblotting of immunoprecipitated production or cell lysates from HEK293 cells overexpressing indicated plasmids. Data are representative of three independent experiments with similar results.

Above data outline the question that how Src increases PI3K activation. The p85 unit of PI3K regulates the PI3K/Akt pathway activation. We found LPS treated *Itgam*^−/−^ peritoneal macrophages exhibited lower p85 phosphorylation and higher protein expression level than that of their littermate control (Fig. 6A). CA-Src overexpression increased the phosphorylation level of p85 in RAW264.7 cells, while CA-Akt could not (Fig. 6B). Cbl family members attach K48-branched polyubiquitin chains, resulting in proteasomal degradation of the decorated client proteins. Cbl-b and c-Cbl were both reported to negatively regulate innate and adaptive immunity^35^. Overexpression and co-IP experiments in HEK293 cells indicated that Src and E3-ligase c-Cbl interacted with p85 (Fig. 6C), while Cbl-b could weakly interact with p85 (data not shown). Furthermore, we found CA-Src could promote the degradation (Fig. 6D) and ubquitination (Fig. 6E) of p85 in a c-Cbl dependent manner. These data indicate that Src promotes p85 degradation and PI3K activation.

### Src inhibition aggravates DSS-induced inflammation and colonic damage *in vivo*

**Figure 7:**
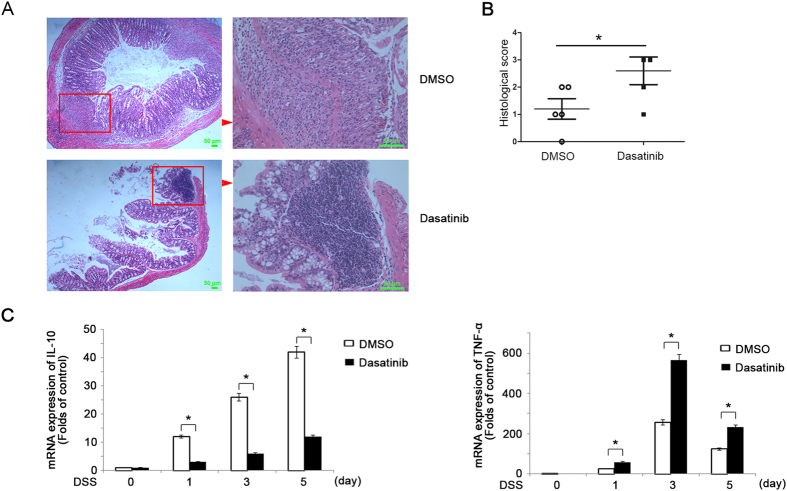
*In vivo* administration of Src inhibitor dasatinib leads to more severe DSS-induced colitis with decrease of IL-10 expression. (**A**,**B**) Hematoxylin-eosin staining (**A**) and the histological score (**B**) of the colon from mice treated with DMSO or dasatinib and fed with 3% DSS in water for 7 days. Magnification: Left: ×50 Right: ×200. (**B**) Q-PCR of IL-10(left) and TNF-α (right) in the colon from mice treated with DMSO or dasatinib after fed with 3% DSS in water. Data are representative of three independent experiments with similar results and presented as means ± SD. *P < 0.01.

Src-kinase inhibitors dasatinib hold great potential as targeted therapy against malignant cells and dasatinib could increase TLR–induced interleukin 12 production in human myeloid cells^36^. Then to further confirm the role of Src in the inflammatory conditions, we investigated function of dasatinib in DSS induced colitis. In accordance with above results, the mice peritoneal injection of dasatinib daily developed more severe DSS-induced colonic damage with more inflammatory cells infiltration (Fig. 7A,B) compared with those treated with DMSO. Accordingly, the infiltration of CD68^+^ cells increased in the dasatinib-treated mice after fed with 3% DSS for 7 days (supplementary Fig. 11).The mRNA expression of IL-10 also decreased, while mRNA of TNF-α increased in the colon of the mice treated with dasatinib (Fig. 7C). The activation of Src and Akt was also impaired in the intestinal macrophages of the dasatinib-treated mice after fed with 3% DSS for 7 days (supplementary Fig. 12B). Meanwhile, *in-vivo* administration of dasatinib did not change the percentage of macrophages in leukocytes (supplementary Fig. 12A). These results further indicate that Src protects mice from DSS-induced colitis via increased induction of IL-10.

## Discussion

The production or the signaling of immune suppressive cytokine IL-10 is tightly related to inflammatory and autoimmune pathologies^1^. Our previous reports demonstrate that CD11b negatively regulates TLR-triggered signaling and responses by promoting Cbl-b medicated TLR adaptors through activating Src-spleen tyrosine kinase (Syk) signal pathway^29^. Now we find that CD11b attenuates DSS-induced colitis by promoting TLR-triggered IL-10 expression via Src-Akt pathway, indicating the critical role of CD11b and its downstream signal Src in maintaining immune homeostasis and controlling TLR-induced innate inflammatory response.

The intestine is continuously exposed to bacterial flora, dietary antigens and potential pathogens. Tightly regulation of PPRs signaling and balance of pro-inflammatory and anti-inflammatory cytokines keep the immune response in check to prevent chronic intestinal inflammation. IL-10 or IL-10R-deficient mice develop inflammatory bowel disease^3^, which requires microorganisms in gut^4^. Recently, IL-10R signaling in macrophages was found to be a key factor driving these innate immunity cells to express homeostatic tolerogenic functions and confirm that CX3CR1^hi^ macrophages are critical players in gut health and inflammation^2^,^37^. Adoptive transfer of peritoneal cells enhances the induction of IL-10 production in colitis^38^. These reports demonstrate the critical role of IL-10 signaling in the second step of immune homeostasis when IL-10 is produced under tightly control. The orchestra of PRRs activation induced pro-inflammatory and anti-inflammatory cytokines, functions as the first step of immune activation during the homeostasis in gut microbiota and innate immunity. Therefore, the proper production of IL-10 in gut remains to be first key step. The discovery of dual function of CD11b-Src signaling in inhibiting pro-inflammatory and promoting anti-inflammatory cytokines production adds new lights on the gut homeostasis of innate immunity.

The function of Src-Syk signal in the regulation of TLR and other PRR signaling are under controversial. Src downstream kinase Syk rescues the increased TLRs signaling in DAP12-deficient macrophages^39^, and Src family member Lyn-deficient mice suffer from myeloid differentiation factor 88 (MyD88)-dependent autoimmune disease^40^, which indicates Src tyrosine kinase signaling functions as a negative regulator for TLR signaling. However the underlying mechanism is unclear. In accordance with the report that CD11b expressed neutrophils produced high amounts of the anti-inflammatory cytokine IL-10 in a DAP12-Syk and MyD88-dependent manner^41^, here we found that CD11b promotes TLR-triggered IL-10 expression via Src-Akt pathway in both macrophages and DCs. Furthermore, we reported for the first time the decreased activation of Src/Akt and increased inflammation in CD11b-deficient macrophages *in vivo*, as shown by isolating macrophages from lamina propria mononuclear cells. Our finding provides further evidence for the negative function of Src family. CD11b was reported to be highly expressed on suppressive cells, such as regulatory DCs and myeloid derived suppressive cells (MDSCs)^42^. However the relationship between CD11b and its suppressive function remains unknown. The function of CD11b and its signaling in IL-10 production also provide potential explanation of the signal mechanism of the correlation between CD11b^high^ and immune suppressive phenotype in regulatory DCs or MDSCs.

Following TLR stimulation, activations of extracellular signal-regulated kinase (ERK) and downstream transcriptor AP1 have a central role in IL-10 expression^1^,^16^,^18^,^43^. Chemical inhibitors of ERK or deficiency of ERK decreases TLR-ligation induced IL-10 production in DCs^17^,^44^. Furthermore, the differences in IL-10 production by macrophages, myeloid DCs and plasmacytoid DCs (pDCs) correlate with the strength of ERK activation in each of these cell types^17^. Following TLR stimulation, ERK is most highly activated in macrophages, with lower activation of ERK in myeloid DCs and the lowest amount of activated ERK in pDCs. PI3K also constrains full immune cell activation by upregulating of the key anti-inflammatory cytokine interleukin 10 and inhibiting of pro-inflammatory cytokines in myeloid immune cells^32^,^33^. Here we found that CD11b-activated Src could increase AP-1 activation by both increasing ERK activation and decreasing the inhibition on AP-1 by GSK3, and CD11b-activated Src on the same time could also inhibit the NF-κB activation. These results provide the novel signal cascade coordination of Src-Akt in regulation of TLR-induced IL-10 production, which also confirm the critical role of AP-1 activation.

In sum, we demonstrate that CD11b-Src plays a critical role in the orchestra of PRRs-induced inflammatory and anti-inflammatory cytokines. In this way, CD11b prevents the pathogenesis of inflammatory immune disorders and contributes to maintenance of homeostasis in gut microbiota and innate immunity.

## Methods

### Mice and Reagents

Mice homozygous for CD11b-deficient mice (B6.129S4-Itgam^tm1Myd^/J; 003991; Jackson Laboratories) were bred in pathogen–free conditions. *Itgam*^−/−^ homozygous mice and *Itgam*^+/−^ heterozygote littermates were derived from the first filial generation mice of *Itgam*^−/−^ mice mating with wide type C57/BL mice. 6–8 weeks of age littermate mice were used in the experiments (body weight and sexuality balanced). All animal experiments were performed in accordance with the National Institute of Health Guide for the Care and Use of Laboratory Animals, with the approval of the Scientific Investigation Board of Second Military Medical University, Shanghai. LPS (O111:B4), poly (I:C) and CpG ODN used were described previously^29^,^45^. PP1 and PP2 were from Calbiochem (San Diego, CA) and Src Ihibitor-1 (Src-I) from Sigma Aldrich (San Diego, CA). Antibodies specific to HA-tag, myc-tag, V5-tag (HRP conjugated) and the agaroses used in immunoprecipitations were from Abcam Inc. (Cambridge, MA). Abs specific for ERK, C-Jun, Src, Akt, p65 and CREB, phospho-specific Abs against Src (Tyr416), p85(Tyr458), Akt (Ser473), ERK (Thr202/Tyr204), C-Jun (Ser73), GSK3α/β (Ser29/Ser9) and GSK3β (Ser9) were from Cell Signaling Technology (Beverly, MA). HRP-conjugated second antibody (TrueBlot) were from eBioscience (San Diego, CA). The Abs against CD45 and F4/80 used in FASC and the antibody against CD68 used in IHC were from Biolegend. The PE anti-phospho-Akt (S473) and ALEXA 488 anti-phospho-Src (Y418) Ab used in FACS was from BD Phosflow. Annexin V was from Biolegend and PI was from Sigma-Aldrich.

### Plasmid constructs and transfection

The recombinant vectors encoding Src (NM_009271), ubiquitin (BC100341), c-Cbl (NM_007619.2), and p85(NM_001024955.2) were constructed by PCR-based amplification from RAW264.7 cDNA and then subcloned into the pcDNA3.1 eukaryotic expression vector (Invitrogen, San Diego, CA) as described previously^29^,^45^. CA-Akt was constructed with a pp-60 c-Src myristoylation sequence in the N-terminus^46^. Transient transfection of plasmids into macrophages with jetPEI reagents (Polyplus-transfection Company, Illkirch, France) was performed following the instructions.

### Cell culture and ELISA assays

RAW 264.7 and HEK293 cell lines were obtained from the American Type Culture Collection. Thioglycolate-elicited mouse peritoneal macrophages were prepared and cultured in endotoxin-free DMEM with 10% FBS (Invitrogen). After the stimulation (100 ng/ml of LPS, 10 μg/ml of poly (I:C) or 0.3 μM of CpG-ODN for all), the concentrations of cytokines IL-10 in the culture supernatants were determined by ELISA kits (R&D Systems, Minneapolis, MN) as described previously 29,45.

### Peritoneal macrophages

6-weeks-old mice were injected with 3% (w/v) Merk thioglycollate medium into the peritoneal cavity of each mouse. 3 days after the injection, the peritoneal macrophages were collected by flushing the peritoneal cavity with RPMI 1640^29^,^45^.

### Bone marrow derived dendritic cells (BMDCs)

Dendritic cells were isolated and culture as previously reports^47^. Briefly bone marrow cells from 6-weeks-old mice were cultured in RPMI 1640 medium with the addition of 10%(v/v) FBS, 20 ng/ml GM-CSF and 10 ng/ml IL-4 for 3 days. The non-adherent cells were gently removed. The remaining cells were cultured for an additional 4–5 days and used as immature BMDCs.

### DSS mice model

6-weeks-old mice were transferred to fresh autoclaved cages and given drinking water supplemented with filter sterilized 3% DSS (w/v)(molecular weight 36,000–50,000 Da; MP Biomedicals LLC) for 7 days, after which the serum and the colon were harvested. The IL-10 in the serum was determined by ELISA. The colon were gently inflated with 1× PBS and kept immersed in fixative for 24 hours before stained with H&E. In addition, the other colon tissues were lysed in TRIzol reagent (Ambion by Life technologies) for RNA extraction.

### DSS mice model with Dasatinib

Dasatinib (Selleck) was dissolved in DMSO at 60 mg/ml and stored in aliquots at −20 °C. On each treatment day, aliquots were thawed and diluted in PBS with 5% polyethylene glycol (PEG-400; Sangon Biotech) and 1% Tween-λ80(Sangon Biotech) immediately before use. Control mice were treated with an equivalent concentration of DMSO dissolved in PBS. Then the diluted Dasatinib (15 mg/kg) or DMSO was injected into the peritoneal cavity of mice every day during the DSS feeding.

### Histological evaluation of DSS-induced colitis

After the hematoxylin–eosin (HE) staining of the colon, the evaluation of the inflammation associated histological changes in the colon was performed with a scale previously described by Stefan Wirtz *et al.*^48^.

### Isolation of Lamina Propria Mononuclear Cells (LPMCs)

Lamina propria mononuclear cells were isolated using a modified protocol^49^. Briefly, colonic tissues were obtained from mice, opened longitudinally, and denuded of the epithelial layer by incubation with Ca^2+^ Mg^2+^ free HBSS/EDTA twice (30 min each) with vigorous shaking at 37 °C. Tissues were washed several times with PBS, minced, and digested at 37 °C twice (1 hour each) with gentle shaking with collagenase IV (Worthington) , DNase I (BBI Life Science) and Dispase II (SIGMA) in the presence of 100 U/mL penicillin and 100 mg/mL streptomycin. The LPMCs were purified by centrifugation through a 35%/80% discontinuous Percoll gradient (GE healthcare).

### RNA quantification

Quantitative real-time RT-PCR analysis was performed by LightCycler (Roche, Basel, Switzerland) and SYBR RT-PCR kit (Takara, Dalian, China) as described previously^29^,^45^. Data were normalized to β-actin expression.

### Immunoprecipitation and Immunoblot

Cells were lysed with RIPA buffer (Cell Signaling Technology, Beverly, MA), or M-PER Protein Extraction Reagent (Pierce, Rockford, IL) supplemented with protease inhibitor cocktail. Protein concentrations of the extracts were measured with BCA assay (Pierce). The immunoprecipitation and immunoblot assays were performed as described previously^29^,^45^.

### Statistical analysis

Results are given as means plus or minus standard deviation (SD). Comparisons between 2 groups were performed using Student’s *t* test. Statistical significance was determined as *P* values less than 0.01.

## Additional Information

**How to cite this article**: Hu, X. *et al.* Integrin CD11b attenuates colitis by strengthening Src-Akt pathway to polarize anti-inflammatory IL-10 expression. *Sci. Rep.* **6**, 26252; doi: 10.1038/srep26252 (2016).

## Supplementary Material

Supplementary Information
