# Author Correction: Integrin CD11b attenuates colitis by strengthening Src-Akt pathway to polarize anti-inflammatory IL-10 expression

**DOI:** 10.1038/s41598-020-76696-w

**Published:** 2020-11-10

**Authors:** Xiang Hu, Chaofeng Han, Jing Jin, Kewei Qin, Hua Zhang, Tianliang Li, Nan Li, Xuetao Cao

**Affiliations:** 1 National Key Laboratory of Medical Molecular Biology and Department of Immunology, Institute of Basic Medical Sciences, Peking Union Medical College, Chinese Academy of Medical Sciences, Beijing, 100730 China; 2 National Key Laboratory of Medical Immunology and Institute of Immunology, Second Military Medical University, Shanghai, China

Correction to: *Scientific Reports*, 10.1038/srep26252, published online 18 May 2016

The data provided in this Article is incomplete.

The original images for the Western blot experiments were not provided. The full-length blots are included in the Supplementary Information file attached to this notice.

## Supplementary information

Supplementary Information.

