# Supplementary material for: Integrin CD11b attenuates colitis by strengthening Src-Akt pathway to polarize anti-inflammatory IL-10 expression
Source: Sci Rep. 2016 May 18;6:26252. doi: 10.1038/srep26252 (PMC4870583; doi:10.1038/srep26252)
Supplement: Supplementary Information [file srep26252-s1.pdf]

Integrin CD11b attenuates colitis by strengthening Src-Akt pathway to polarize anti-inflammatory IL-10 expression

Xiang Hu[1,2,3], Chaofeng Han[2,3,*], Jing Jin[2], Kewei Qin[2], Hua Zhang[2], Tianliang Li[2], Nan Li[1], and Xuetao Cao[1,2,*]

[1] National Key Laboratory of Medical Molecular Biology & Department of Immunology, Institute of Basic Medical Sciences, Peking Union Medical College, Chinese Academy of Medical Sciences, Beijing 100730, China

[2]National Key Laboratory of Medical Immunology & Institute of Immunology, Second Military Medical University, Shanghai, China;

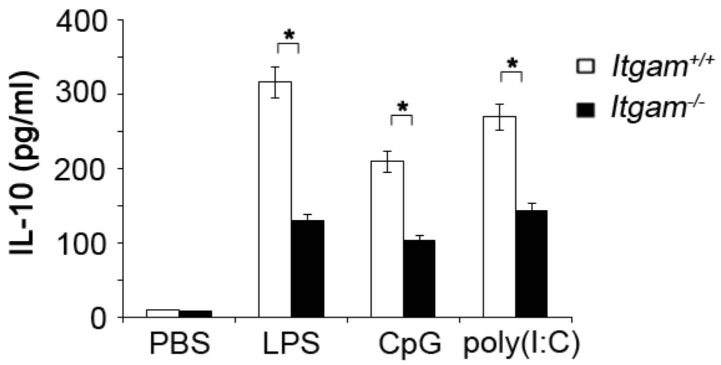

Supplementary Fig. 1 ELISA of IL-10 production in supernatant from *Itgam*$^{+/+}$ or *Itgam*$^{-/-}$ peritoneal macrophages(6×10^5) stimulated with LPS (100ng/ml), CpG (0.3 μM) or poly(I:C) (10 μg) for 12h. Data are presented as means ± SD. *, P<0.01.

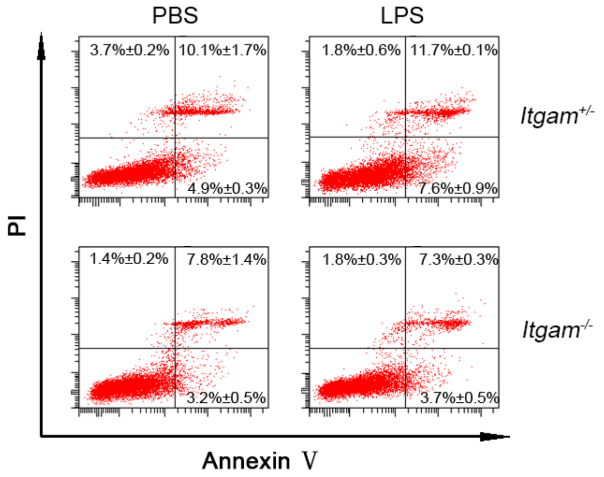

Supplementary Fig. 2 The peritoneal macrophages from *Itgam*$^{+/-}$ and *Itgam*$^{-/-}$ mice were analyzed by FACS by staining with Annexin Ⅴ and PI after stimulated with PBS or LPS for 6 hours. The Annexin Ⅴ positive cells were regarded as apoptotic cells. A representative experiment is presented and the statistic analysis was performed by three experiments.

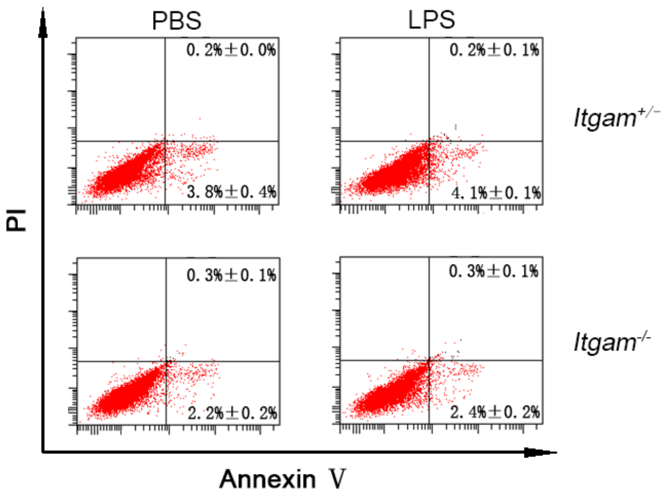

Supplementary Fig. 3 The BMDCs from *Itgam*$^{+/-}$ and *Itgam*$^{-/-}$ mice were analyzed by FACS by staining with Annexin Ⅴ and PI after stimulated with PBS or LPS for 6 hours. The Annexin Ⅴ positive cells were regarded as apoptotic cells. A representative experiment is presented and the statistic analysis was performed by three experiments.

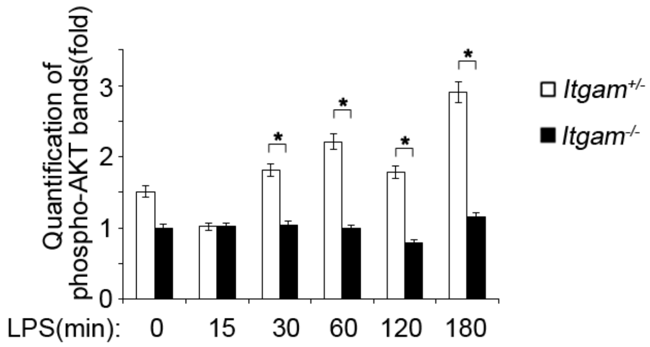

Supplementary Fig. 4 The quantification of the phospho-Akt bands in figure 3C was analyzed by Tanon Gel Image System. Data are presented as means ± SEM. *, $P<0.01$.

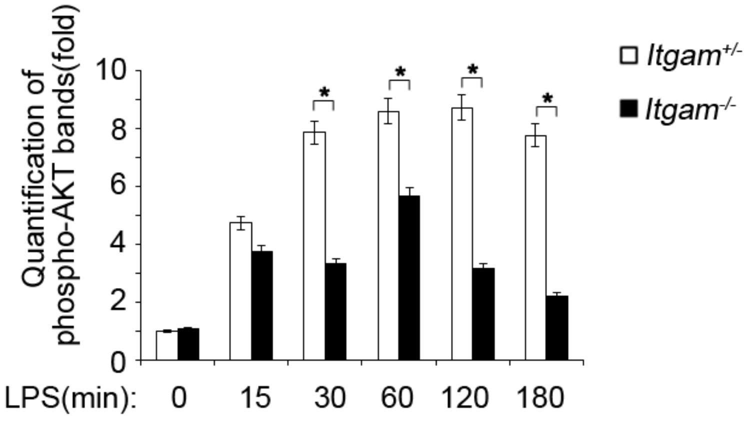

Supplementary Fig. 5 The quantification of the phospho-Akt bands in figure 3D was analyzed by Tanon Gel Image System. Data are presented as means ± SEM. *, P<0.01.

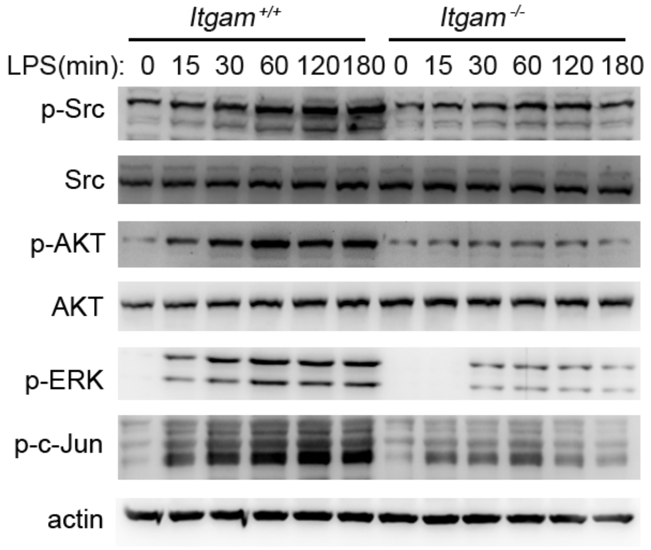

Supplementary Fig 6. Immunoblotting of the cell lysates from *Itgam*$^{+/+}$ or *Itgam*$^{-/-}$ peritoneal macrophage(6×10^5) for the indicated time with indicated antibodies.

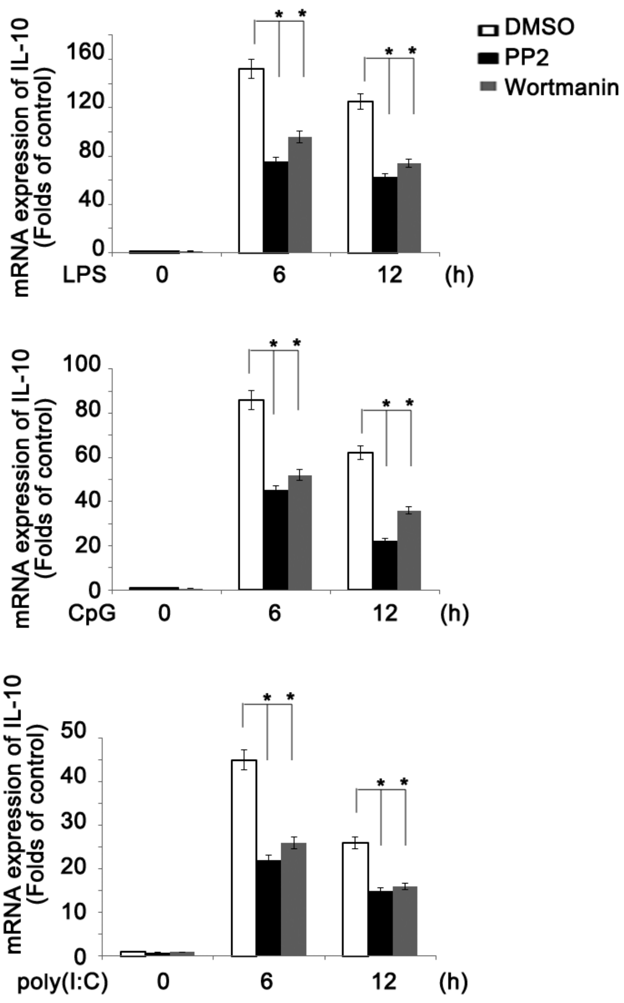

Supplementary Fig. 7 Q-PCR of IL-10 in WT peritoneal macrophages(6×10^5) pretreated with PP2 (10μM) or Wortmanin (5μM) for 30 mins and then stimulated with LPS (100 ng/ml), poly(I:C) (10μg/ml), or CpG-ODN (0.3 μM) for 6h or 12h. Data are presented as means ± SD. *, P<0.01.

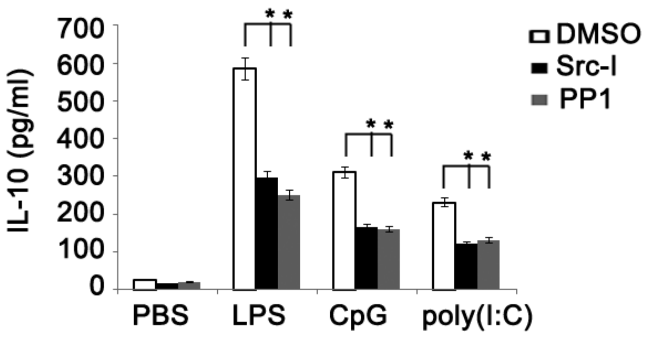

Supplementary Fig. 8 ELISA of IL-10 in supernatant from WT peritoneal macrophages(6×10^5) pretreated with Src-I (10μM) or PP1 (10μM) for 30 mins and then LPS (100 ng/ml), poly(I:C) (10μg/ml), or CpG-ODN (0.3 μM) for 24h. Data are presented as means ± SD. *, P<0.01.

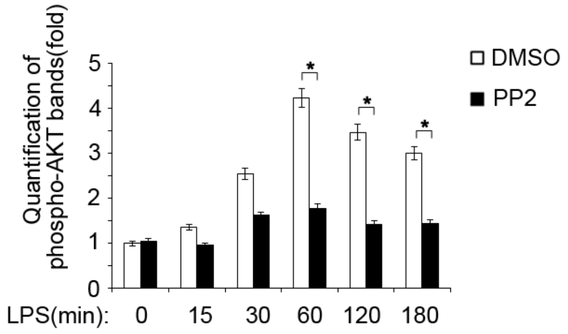

Supplementary Fig. 9 The quantification of the phospho-Akt bands in figure 4C was analyzed by Tanon Gel Image System. Data are presented as means ± SEM. *, P<0.01.

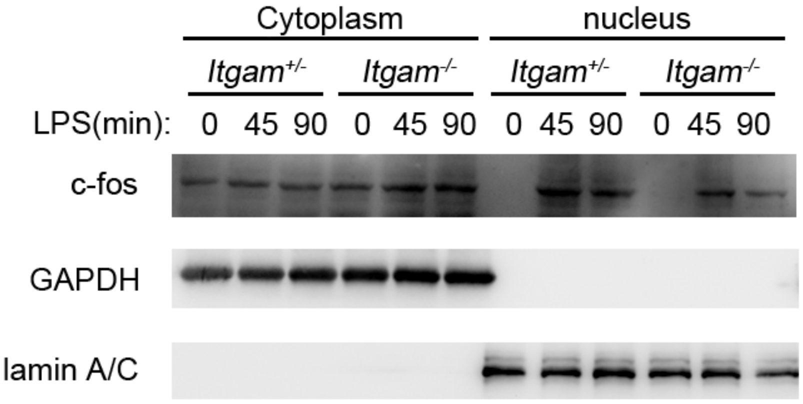

Supplementary Fig. 10 Immunoblotting of cytoplasmic or nuclear extracts from *Itgam*$^{+/-}$ or *Itgam*$^{-/-}$ macrophages stimulated with LPS (100 ng/ml) for indicated time with indicated antibodies.

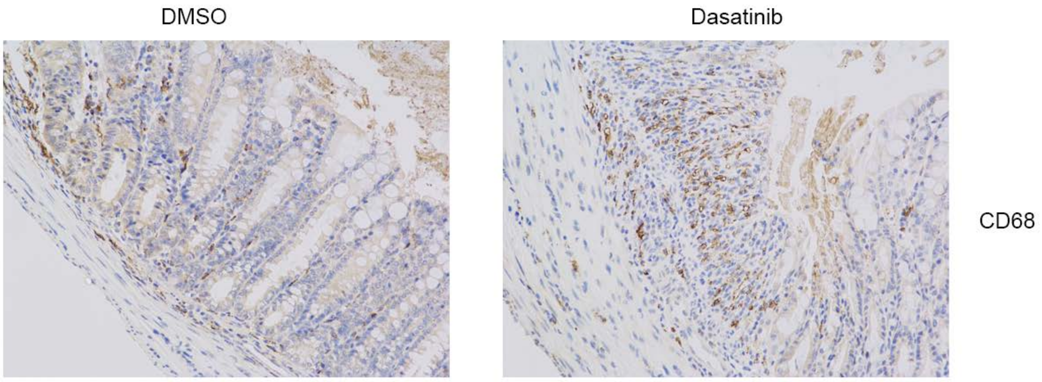

Supplementary Fig. 11 Immunohistochemistry analysis of CD68 expression in the colon from mice treated with DMSO or dasatinib and fed with 3% DSS in water for 7 days with anti-mouse CD68 Abs. Magnification: ×200.

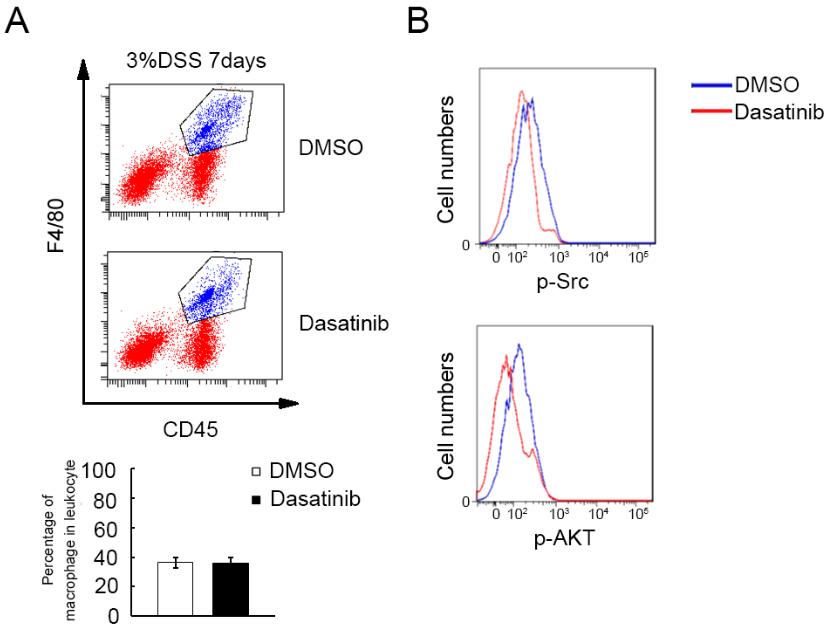

Supplementary Fig. 12(A, B) The percentage of intestinal macrophages(CD45$^+$F4/80$^+$) in leukocytes(CD45$^+$) (A) and the expression level of phospho-Src and phospho-Akt of the CD45$^+$F4/80$^+$ cells (B) from the colon of mice treated with DMSO or dasatinib and fed with 3% DSS in water for 7 days were analyzed by FACS. A representative experiment is presented and the statistic analysis was performed by three experiments.
