# Supplementary material for: Author Correction: Integrin CD11b attenuates colitis by strengthening Src-Akt pathway to polarize anti-inflammatory IL-10 expression
Source: Sci Rep. 2020 Nov 10;10:19885. doi: 10.1038/s41598-020-76696-w (PMC7655800; doi:10.1038/s41598-020-76696-w)

Integrin CD11b attenuates colitis by strengthening Src-Akt pathway to polarize anti-inflammatory IL-10 expression

Xiang Hu[1,*], Chaofeng Han[2,*], Jing Jin[2], Kewei Qin[2], Hua Zhang[2], Tianliang Li[2], Nan Li[2] &Xuetao Cao[1,2]

1National Key Laboratory of Medical Molecular Biology & Department of Immunology, Institute of Basic Medical Sciences, Peking Union Medical College, Chinese Academy of Medical Sciences, Beijing 100730, China.

2National Key Laboratory of Medical Immunology & Institute of Immunology, Second Military Medical University, Shanghai, China.

*These authors contributed equally to this work. Correspondence and requests for materials should be addressed to

C.H. (email: hcf@immunol.org) or X.C. (email: caoxt@immunol.org)

Uncropped data for Figure3

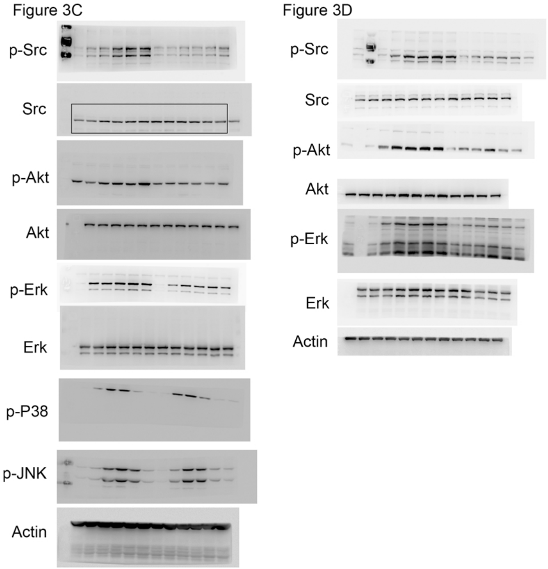

Uncropped data for Figure4

Figure 4C

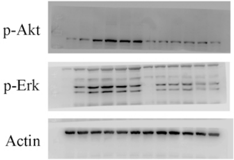

Figure 4D

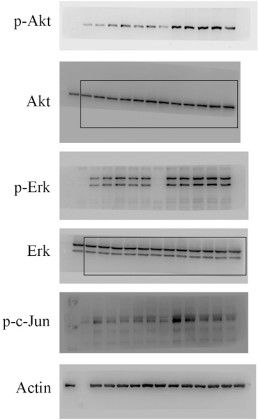

Uncropped data for figure 5

Figure 5A

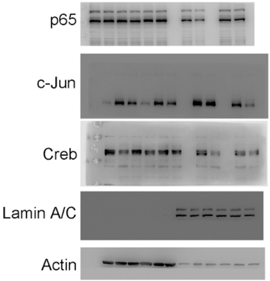

Figure 5B

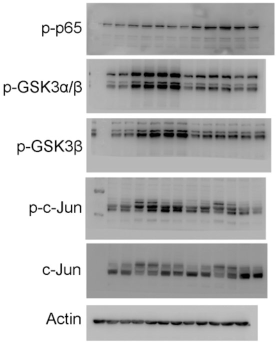

Figure 5C

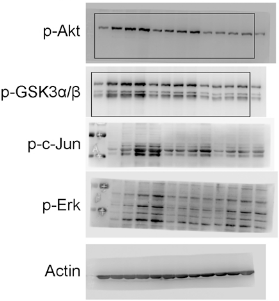

Figure 5D

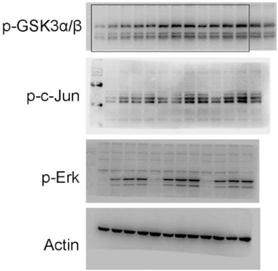

Figure 5E

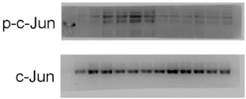

Figure 5F

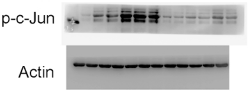

Uncropped data for Figure 6

Figure 6A

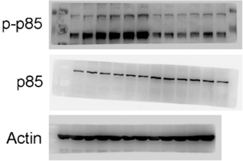

Figure 6D

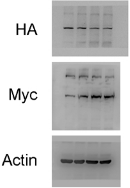

Figure 6B

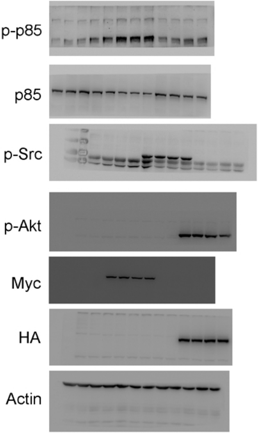

Figure 6C

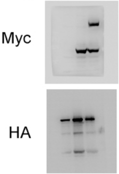

Figure 6E

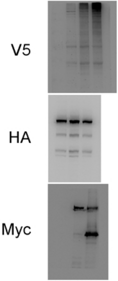

Uncropped data for supplymentary data

S.Fig. 6

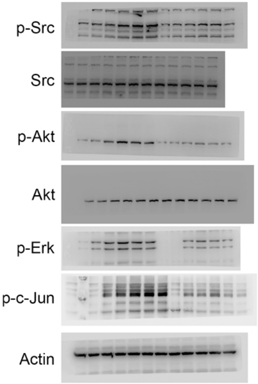

S.Fig.10

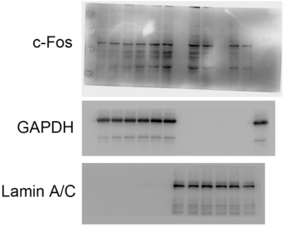

Supplement: Supplementary file 1 — Supplementary Information. [file 41598_2020_76696_MOESM1_ESM.pdf]
